# A New Construction of High Performance LDPC Matrices for Mobile Networks

**DOI:** 10.3390/s20082300

**Published:** 2020-04-17

**Authors:** Moein Sarvaghad-Moghaddam, Waheed Ullah, Dushantha Nalin K. Jayakody, Sofiène Affes

**Affiliations:** 1 Quantum Design Automation Lab, Amirkabir University of Technology, Tehran 9821, Iran; moeinsarvaghad@aut.ac.ir; 2 School of Electrical and Information Engineering, University of the Witwatersrand, WITS 2050, South Africa; waheed.ullah@wits.ac.za; 3 School of Computer Science and Robotics, National Research Tomsk Polytechnic University Tomsk, 634050 Tomsk, Russia; 4 Centre Énergie, Matériaux et Telecommunications, Institut National de la Recherche Scientifique, Montreal, QC H5A 1K6, Canada; affes@emt.inrs.ca

**Keywords:** LDPC, regular parity check matrix, IoT, mobile networks, quasi-cyclic (QC) LDPC

## Abstract

Secure and reliable information flow is one of the main challenges in social IoT and mobile networks. Information flow and data integrity is still an open research problem. In this paper, we develop new methods of constructing systematic and regular Low-Density Parity-Check Matrices (LDPCM), inspired by the structure of the Sarrus method and geometric designs. Furthermore, these codes have cyclic structure and therefore, are less complex in computation and also require less memory in hardware implementation. Besides, an optimal method of post-processing for deleting girths four is presented. Numerical results show that the codes constructed by these methods perform well over the additive white Gaussian noise (AWGN) channel when decoded with the sum-product LDPC iterative algorithms. The proposed methods can be very efficient in terms of reducing memory consumption and improving the convergence speed of the decoder particularly in IoT and mobile networks.

## 1. Introduction

In the recent decade, we have seen significant growth in the use of smart devices in different applications such as IoT [1]. The most important criteria of the social Internet of Things (IoT) are scalability, trust, and discovery of the resources, social computing, information flow analysis, and data integrity. Data reliability and integrity assure the accuracy and completeness of data sent and received [2]. In social IoTs and mobile networks, data reliability is often at risk due to distortion, Gaussian noise and attenuation during wireless transmission of data; it means that we are not able to decode the data correctly to get the right message. To make sure the data reliability and integrity in the presence of noise, error correction codes can be used.

Low-Density Parity-Check (LDPC) codes play a vital rule in today wireless and wired communications and are currently the most powerful coding technique to achieve near-Shannon-capacity performance [3] for a wide range of noisy channels. LDPC codes were first invented by Gallager in 1962 [4] and later discovered by Mackay in 1996 [5,6]. Though forgotten for three decades, LDPC codes gained attention recently and substantial research work has been done in designing the parity check matrices, low complexity encoding and decoding algorithms and numerous practical applications [7,8,9,10,11,12].

The bit error performance of the LDPC code primarily depends on the construction [13] of LDPCM and on parameters such as row and column weights, rate, girth, and code size. Constructions of LDPCM can be categorized into two broad groups: random (irregular) and algebraic constructions (regular). The computer search method is generally used for random construction of LDPCM with a predefined set of design rules based on the Tanner graphs [14] which gives connectivity of the bit (variable) nodes and the check nodes. Randomly constructed irregular LDPC codes can perform within 0.0045 dB of Shannon limit [15] but these codes are very complex to implement in hardware. Methods of algebraic construction are used to construct cyclic or quasi-cyclic LDPC codes with combination methods. This structured LDPC codes [16,17,18], in general, are simple to encode and decode as compared with the random codes. The LDPC codes have shown the capacity approaching performance, but at the expense of high complexity which is the main hurdle to be adopted, these codes in many real-world applications and implementations. Recently LDPC codes have been adopted by digital video broadcasting and IEEE standards like DVB-S2, DVB-X2, WiFI and WiMax [19,20].

### 1.1. Case Study

#### 1.1.1. LDPC User Cases in 5G

Cellular networks and the Internet of Things are the main market drivers for 5G and beyond. There is a large number of use cases for cellular networks and the Internet of Things, such as virtual reality, augmented reality and remote sensing, eHealth services, automotive driving and many more.

Keeping in view the IoT enabled devices, 5G is meant to operate at higher speeds and make the delay nearly non-existent, giving way to a seamless information flow [21]. Mobile IoT-based devices offer the low-cost, low-power consumption solution in comparison with the existing 4G. This 5G enabled mobile network will improve coverage compared with existing wide-area technologies, will offer secure connectivity, authentication and network scalability for capacity enhancement [22]. For user data, 5G channel codes, similar to a 4G system, should also support a variable code rate and length for both control information and user data as well as hybrid automatic repeat request. During the standardization process of 5G [23], several coding schemes based on the aforementioned requirements are considered and LDPC coding has been adopted for user data focusing on low latency in 5G cellular communications.

To achieve high data throughput in 5G, parallelism in encoding and decoding plays an important role. Systematic structured LDPC codes naturally keep parallelism in encoding and decoding, and high data rate encoder and decoder can be realized by such parallelism. Adaptive rate compatibility to select an arbitrary length of transmitted codeword bits from parental code output and a variable code length are other important functionalities of 5G channel codes, and the 5G LDPC code design show such functionalities. There are some recent coding scheme [24] recommended for high throughput 5G networks as a subfield of network coding, which is of particular interest as well.

#### 1.1.2. LDPC User Cases in IoT

The fundamental question to the design of the physical layer for IoT is simply the trade-off between performance (information processing latency, throughput, bit error rates, etc.) vs. computational complexity and some required overhead (e.g., frame overhead, feedback ). In the present cellular system design up to LTE 4G, the main concern is the high data rate downlink for data communication having large packet sizes. The demand for over increasing data rates with limited spectrum resources has ultimately led to the development of communication physical layer that can better exploit the wireless channel and can correct the errors that occurred during transmission using error correction techniques like LDPC [25]. Energy conservation and consumption are the major constraints in IoT and WSNs and, therefore, reducing the number of the packets in error to re-transmit is very important. Other than the energy harvesting technique, one efficient way to reduce the energy consumption using Forward Error Correction (FEC) codes. Bearing in mind this requirement, we propose matrices to enhance the ability of LDPC error correction and detection.

#### 1.1.3. Would LDPC Be a Candidate for 6G

The 6G communication network channel-coding technique should have features such as low-complexity, high coding gain, low-latency, high-throughput, and very flexible code parameters and this can be filled with the choice of LDPC codes. Currently, the 5G communication network has been standardized, but yet to come into the market, but greed for high date rate and fast wireless communication is still strong. There is a growing demand for increased spectrum and power resources. Therefore, further improvement in transmission technology needs to be updated to achieve the rapid growth of the wireless communication capacity [26]. The beyond 5G mobile communication (6G) should perform well in terms of wireless coverage and user experience. To overcome these challenges, we need to use an improved error-correcting code for efficient transmission capabilities of 6G. Among the error correction and detection techniques, LDPC coding can significantly improve the reliability of communication systems.

#### 1.1.4. Potential Applications of the Proposed Scheme

One of the basic features in 5G and IoT is the variation of data rate. We might have a huge date flow or as low as few bits. Most of the error correction codes to perform better for longer code-word length and show performance degradation for the small code-word length. The LDPC decoding algorithm’s error correction capability mainly depends on the code-word length, and the design of the parity check matrix. Normally, the LDPC decoders [5] perform better with a larger code-word and with well designed parity-check matrices. Looking at the requirement and the constraints of the future communication systems (5G and beyond, IoTs), we proposed medium and small size regular parity check matrices for completely fulfilling the mentioned requirements. The proposed matrices for LDPC codes perform better than existing small and medium-size matrices and therefore are more suitable for such applications.

### 1.2. LDPC Code Structure

Today a lot of techniques have been developed to design and develop high-performance LDPC codes [5,15,27,28,29,30]. Most of the high-performance LDPC codes are generated randomly by the computer search and lacking the algebraic structure. These types of LDPC codes are hard to analyze theoretically as well as to simply implement. Good LDPC codes usually have a very long code length, which causes a high computational complexity and requires large hardware memory to store the matrix at both the encoder and decoder. In decoding an LDPC code with the Sum-Product Algorithm (SPA), the bit error (BER) performance depends on cycles of short lengths [6,28,31] in the Tanner graph. It has been proven by research that the shorter cycles, generally of length 4, makes the decoding bit error rate (BER) very poor because it makes the decoding iterations much correlated. LDPC codes show better performance for long block length, which makes them difficult for resource-constrained hardware implementation. However, decoding of LDPC codes with short constraint length can be performed over short windows resulting in a very good performance. This technique is known as the sliding window LDPC decoder [32].

The binary LDPCM is used to encode and decode the sequence information. Consider a (k×n) binary generator matrix *G*, where *k* is the length of message bits, there exists an ((n−k)×n) binary LDPCM H such that ${\mathrm{GH}}^{T}=0$ where $H^{T}$ is the transpose of H and 0 is a k×(n−k) all zero matrix. In a systematic form, LDPCM *H* can be written in the form:

(1) $$H=[I_{n-k}:P^{T}]$$

Let $h_{1}$, $h_{2}$, $h_{3}$,........, $h_{J}$ in being the rows of H

(2) $$h_{z}=\left(h_{z,1},h_{z,2},\ldots \ldots ,h_{z,n}\right)\;for\;1\leq z\leq Z.$$

An n-tuple $v=(v_{1},v_{2},\ldots \ldots ,v_{n})$ is a codeword specified by H if and only if the inner product shown in Equation (3) be zero.

(3) $$S_{z}=v.h_{z}=\sum_{l=0}^{n}\;v_{l}\;h_{z,l}=0.$$

Equation (3) gives the syndrome for that particular parity check equation sum based on module 2. The LDPC code structure is defined characteristically as $\left(n,w_{c},w_{r}\right)$ where $w_{r}$ is the number of ones (1s) in a row of a binary parity check matrix, $w_{c}$ is the number of ones (1s) in a column of a parity check matrix and *n* is the length of the code-word equal to the number of the column in a parity check matrix. To design the regular LDPCM, the following condition must be held:

(4) $$m.w_{r}=n.w_{c},$$

where H is the sparse binary parity check matrix and the code-word is obtained from the generator matrix (G) and information bits. The LDPCM *H* has been shown in Equation (5), with $n=8,w_{c}=2$ and $w_{r}=4$.

$$\;b_{1}\;b_{2}\;b_{3}\;b_{4}\;b_{5}\;b_{6}\;b_{7}$$

(5) $$H=\left[\begin{array}{cccccccc} 1 & 1 & 1 & 1 & 0 & 0 & 0 & 0\\ 0 & 0 & 0 & 0 & 1 & 1 & 1 & 1\\ 0 & 1 & 0 & 0 & 1 & 1 & 0 & 1\\ 1 & 0 & 1 & 1 & 0 & 0 & 1 & 0 \end{array}\right]$$

**Figure 1:**
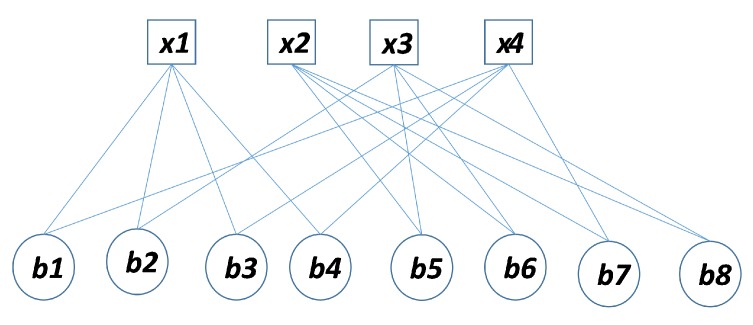
Matrix H Tanner representation.

The sparse binary parity check matrix can be represented by a Tanner graph [14]. Rows of the parity check matrix show bit (variable) nodes and columns of the parity check matrix shows check nodes and the 1s(ones) in a row and column give the connections between bit nodes and check nodes. The set of bit nodes connecting to check nodes and the set of the check nodes connecting to bit node is illustrated as $n\left(j\right)=h_{ji}=1$ and $m\left(i\right)=h_{ji}=1$ respectively where j=1,2,3,……,m and i=1,2,3,……,n. The H matrix in Equation (5) has the Tanner graph illustrated in Figure 1 and Equation (6). The algebraic form of check nodes and variable nodes can be given as below:

(6) $$\begin{array}{cc} x_{1} & =b_{1}+b_{2}+b_{3}+b_{4}\\ x_{2} & =b_{5}+b_{6}+b_{7}+b_{8}\\ x_{3} & =b_{2}+b_{5}+b_{6}+b_{8}\\ x_{4} & =b_{1}+b_{3}+b_{4}+b_{7} \end{array}$$

**Figure 2:**
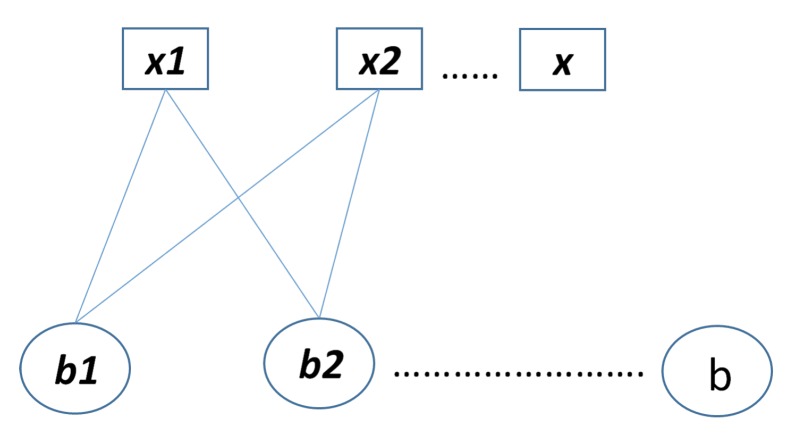
Example of a 4-cycle.

Mackay et al. [5,6] discovered that short cycles tend to degrade the performance of the LDPC decoder. To achieve good performance, it is highly recommended to remove short cycles, especially 4 cycles in the construction of the LDPC parity matrix. Four cycles are illustrated as a tanner graph in the following Figure 2 as a general example.

In the papers [16,18,33], some methods for memory-efficient construction of parity check matrices have been demonstrated. Gallagar, in his paper [4] has introduced a specific construction method for regular LDPC codes as shown in Equations (7) and (8). In Gallager’s method, the transpose of regular LDPC $\left(n,w_{c},w_{r}\right)$ matrix *H* has the form of Equation (7).

(7) $$H^{T}=\left[H_{1}^{T},H_{2}^{T},\ldots \ldots ,H_{Wc}^{T}\right].$$

The matrix $H_{1}$ has *n* columns and $\frac{n}{wr}$ rows. The $H_{1}$ contains a single 1 in its each column and contains 1’s in its $i_{th}$ row from column $\left(i-1\right)w_{r}+1$ to column $i_{wr}$. Random permutation of the columns of $H_{1}$ with equal likelihood, the next matrices $H_{2}$ to $H_{wc}$ are developed. The regular LDPCM H constructed by Gallager’s methods with parameters $\left(n=20,w_{c}=3,w_{r}=4\right)$ is given by Equation (8).

(8) $$H=\left[\begin{array}{cccccccccccccccccccc} 1 & 1 & 1 & 1 & 0 & 0 & 0 & 0 & 0 & 0 & 0 & 0 & 0 & 0 & 0 & 0 & 0 & 0 & 0 & 0\\ 0 & 0 & 0 & 0 & 1 & 1 & 1 & 1 & 0 & 0 & 0 & 0 & 0 & 0 & 0 & 0 & 0 & 0 & 0 & 0\\ 0 & 0 & 0 & 0 & 0 & 0 & 0 & 0 & 1 & 1 & 1 & 1 & 0 & 0 & 0 & 0 & 0 & 0 & 0 & 0\\ 0 & 0 & 0 & 0 & 0 & 0 & 0 & 0 & 0 & 0 & 0 & 0 & 1 & 1 & 1 & 1 & 0 & 0 & 0 & 0\\ 0 & 0 & 0 & 0 & 0 & 0 & 0 & 0 & 0 & 0 & 0 & 0 & 0 & 0 & 0 & 0 & 1 & 1 & 1 & 1\\ 1 & 0 & 0 & 0 & 1 & 0 & 0 & 0 & 1 & 0 & 0 & 0 & 1 & 0 & 0 & 0 & 0 & 0 & 0 & 0\\ 0 & 1 & 0 & 0 & 0 & 1 & 0 & 0 & 0 & 1 & 0 & 0 & 0 & 1 & 0 & 0 & 0 & 1 & 0 & 0\\ 0 & 0 & 1 & 0 & 0 & 0 & 1 & 0 & 0 & 0 & 1 & 0 & 0 & 0 & 1 & 0 & 0 & 0 & 1 & 0\\ 0 & 0 & 0 & 1 & 0 & 0 & 0 & 0 & 0 & 0 & 1 & 1 & 0 & 0 & 0 & 1 & 0 & 0 & 0 & 1\\ 0 & 0 & 0 & 0 & 0 & 0 & 0 & 0 & 1 & 0 & 1 & 0 & 0 & 0 & 0 & 0 & 1 & 0 & 1 & 1\\ 0 & 1 & 0 & 0 & 0 & 0 & 0 & 1 & 0 & 1 & 1 & 0 & 0 & 0 & 0 & 0 & 1 & 0 & 1 & 1\\ 1 & 0 & 1 & 0 & 0 & 1 & 1 & 0 & 0 & 0 & 1 & 0 & 0 & 0 & 0 & 0 & 1 & 0 & 1 & 1\\ 1 & 0 & 1 & 0 & 0 & 0 & 0 & 0 & 0 & 1 & 1 & 0 & 0 & 0 & 0 & 0 & 1 & 0 & 1 & 1\\ 0 & 0 & 0 & 0 & 1 & 0 & 1 & 0 & 1 & 0 & 1 & 0 & 0 & 0 & 0 & 0 & 1 & 0 & 1 & 1\\ 0 & 1 & 0 & 1 & 0 & 1 & 0 & 0 & 1 & 0 & 1 & 0 & 0 & 0 & 0 & 0 & 1 & 0 & 1 & 1 \end{array}\right]$$

**Figure 3:**
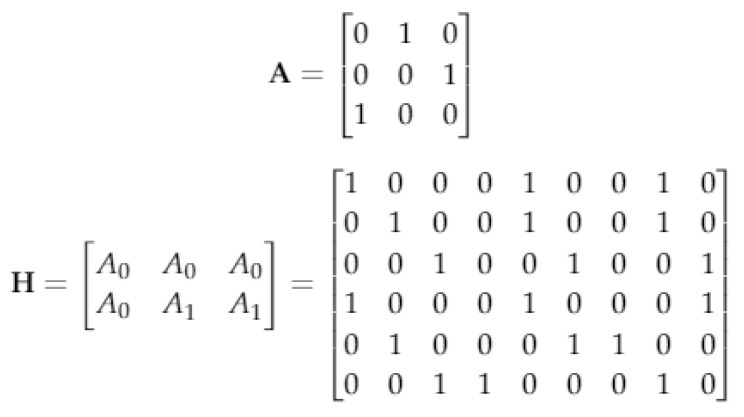
Illustration of the binary LDPCM H for $\left(w_{c}-1\right)\left(w_{r}-1\right)=2$ in algebraic construction.

In [34,35], an algebraic method for design of the binary LDPCM H has been presented. In this method, defining $A^{0}=I_{a}$ the LDPCM H can be constructed as Equation(9).

(9) $$H=\left[\begin{array}{ccccc} A^{0} & A^{0} & A^{0} & \ldots & A^{0}\\ A^{0} & A^{1} & A^{2} & \ldots & A^{wr-1}\\ A^{0} & A^{2} & A^{4} & \ldots & A^{2(wr-1)}\\ A^{0} & A^{wc-1} & A^{2(wc-1)} & \ldots & A^{\left(wc-1)(wr-1\right)} \end{array}\right]$$

where $A_{i}$ is the matrix formed after shifting each rows of the identity matrix $I_{a}$ in a cyclic way by *i* position from left to the right. The matrix H shown in Equation (9) has $w_{c}$ rows and $w_{r}$ columns, and it is a regular $\left(w_{r}a,w_{c},w_{r}\right)$ with the equal number of $w_{r}$ and $w_{c}$ in each row and column respectively. The construction method in Equation (9), gives a 4-cycle free matrix. For example, in Figure 3, construction of *H* matrix with $w_{c}=2$ and $w_{r}=3$ using the algebraic construction method is shown.

In this paper, two methods for designing regular LDPCM are introduced. The first method is inspired by the structure of the Sarrus rule in the mathematic [34,35]. This involves the calculation of the determinant of 3×3 matrix and another method is based on an algebraic construction. Furthermore, a post-processing method is introduced for achieving all possible states in creating regular parity check matrices. Based on the post-processing method, an algorithm for removing the girths 4 in the regular parity check matrices is introduced. In addition, two mathematical methods for removing girths 4 and 6 are presented. This paper is divided into sections as follows: in Section 2, the proposed methods are explained. In Section 3, algorithms for removing girths 4 and 6 in regular parity check matrices are proposed. In Section 4 performance evaluation and comparison are presented. This is followed by the conclusion in Section 5.

### 1.3. Standards Including LDPC Codes and Recent Protograph Codes

LDPC codes are included in different standards for wired and wireless data transmission. ITU-T G.9960 [36] recommends the LDPC Codes system architecture for wire-line based home networking. The recommended coding scheme defines the systematic quasi-cyclic (QC) LDPC encoder followed by a puncturing block. The consultative Committee for Space Data Systems(CCSDS) [37] also included recently LDPC codes for deep space telemetry and near-Earth. QC LDPC codes composed of an array of circulant permutation matrices of size *b* × *b*, are recommended by CCSDS fro deep-space telemetry where the size of *b* corresponds to the information block length *k*. For digital communication, LDPC codes are included in various additional standards [38], specifically:

DVB-S2: The second-generation digital video broadcasting fro satellite application recommends LDPC codes concatenated with BCH codes. The code length is 64800 for various code rate of 1/4,1/3,2/5,1/2,3/5,2/3,3/4,4/5,5/6,8/9,and9/10.

WiMedia: For short-range, high data rates applications, WiMedia recommends LDPC as the forward correction code (FEC).

IEEE802.11-2012: This standard also recommend LDPC codes as an optional choice.

The protograph based LDPC codes comprise relatively few nodes and edges and are specifically suited for reliable optical communication systems [39,40]. These codes can achieve capacity-approaching performance with a high code rate. An extension to these codes for block fading channels are known as Root-Photographs LDPC Codes [41].

## 2. Proposed Methods for Construction of Parity Check Matrix

**Figure 4:**
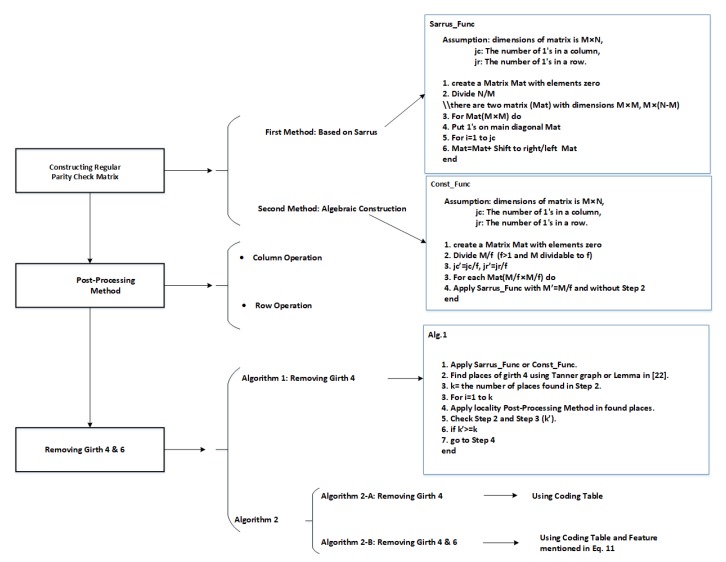
A flowchart for explaining the proposed methods with some pseudocode.

In this section, two methods for the construction of regular parity check matrices are presented. Moreover, a post-processing method for arriving at all possible states is proposed. In the next section, two methods are presented for removing girth 4. Figure 4 shows a flow chart and some of the quasi codes for methods mentioned in this section.

### 2.1. Method 1: Sarrus-Based Method

In this sub-section, first, a background of the Sarrus method for calculating the determinants of matrices 3×3 is explained then based on this method, the first proposed method is explained.

#### 2.1.1. Background

Sarrus method is used for computing the determinants of matrices of order three [34,35]. By using this method [6,14,16,27], as shown in Equation (10), we have to extend the matrix, so that we can calculate the successive diagonals from upper left to bottom right by multiplying the values. The results are summed and the same calculation is done with the diagonals from upper right to bottom left. The difference between the two sums is the determinant of the matrix, although it is not necessary to know this method with details and we only use the topology of structure and relations of elements used in this method.

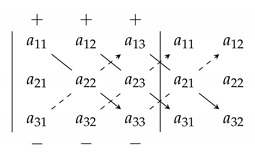
(10)

Arithmetic expression for calculating the determinant of the matrix is as Equation (11):

(11) $$detA=\left|\begin{array}{ccc} a_{11} & a_{12} & a_{13}\\ a_{21} & a_{22} & a_{23}\\ a_{31} & a_{32} & a_{33} \end{array}\right|$$

$$\begin{array}{cc} \; & =a_{11}a_{22}a_{33}+a_{12}a_{23}a_{31}+a_{13}a_{21}a_{32}\\ \; & -a_{31}a_{22}a_{13}-a_{32}a_{23}a_{11}-a_{33}a_{21}a_{12} \end{array}$$

**Figure 5:**
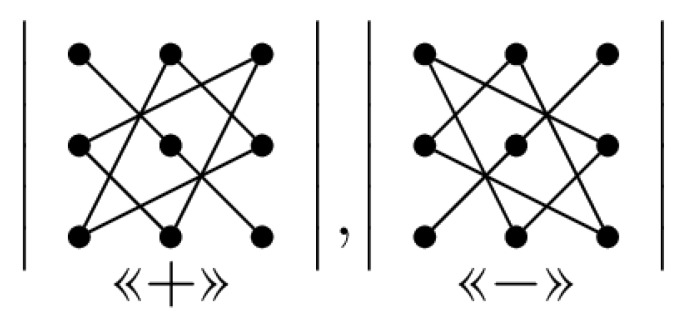
Illustration of using of method 1 for putting ones in matrix 3 × 3.

The Sarrus method can be done as a triangle’s rule [42] as shown in Figure 5. In this method, the product of diagonal elements and product of elements in both vertex of two triangles of the first determinant get the “+” sign and the product of diagonal elements and product of elements in the both vertex of two triangles of the second determinant get the “−” sign.

The idea in the first proposed method is based on triangular specified places and using of topology shown in Figure 5 that these places are used to put ones and the creation of regular parity check matrices.

#### 2.1.2. The first Proposed Method

**Figure 6:**
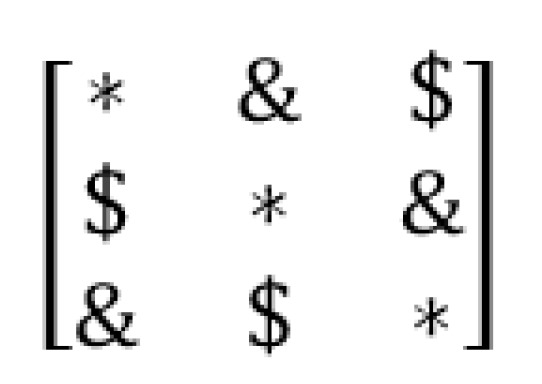
Illustration of using of method 1 for putting ones in matrix 3×3.

The first method is inspired by the structure and topology used in the triangular Sarrus method. As shown in Figure 5, the number of 1’s required in each row and column, can be placed in positions determined in matrix (as triangular in dot placements), i.e., first, to place ones in the main diagonal (ones in position of the symbol “*” in Figure 6, a matrix with only 1 in each row and column is created (identity matrix), then needed other ones are placed as triangular (ones in position of the symbol “&” or symbol “$” in Figure 6.

**Figure 7:**
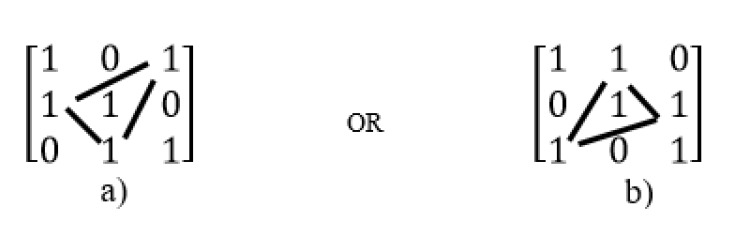
Applying the first proposed method to a 3×3 matrix with two ones in each row and column. (**a**) the first possible state, (**b**) the second possible state.

In other words, in triangular method, other ones (except ones placed in the main diagonal of the matrix) place as diagonal feature so that the number of ones in each diagonal must be equal to the number of elements on the main diagonal (or the number of elements in each row), e.g., if the number of ones in each row is 2 and dimensions of matrix is 3×3, then after forming of the identity matrix, reminder ones can be placed as the states shown in Figure 7.

**Figure 8:**
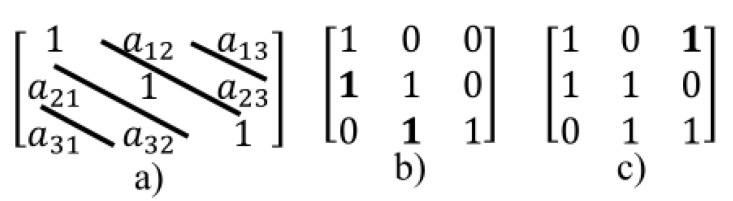
Explanation with different view for the first proposed method in Figure 7a step by step (**a**–**c**). (**a**) operation for the example illustrated in Figure 7a, (**b**) putting ones in it, (**c**) another side.

In the Figure 7, the matrix dimension is 3×3 and the number of elements on the main diagonal (the number of rows) is 3. Therefore, the number of ones will place as diagonal must be 3. The remaining elements are added to the two sides of the main diagonal of the matrix as triangular. For example, if elements in places (2, 1) and (3, 2) in Figure 7 (that the first and second numbers show the number of rows and columns, respectively) are selected to put ones as diagonal. Then, according to triangular rule, the number of ones in this matrix must be three, So, the place (1,3) must be selected for putting one. In other words, if a matrix is divided into parts as diagonal so that the number of parts is equal to the number of rows ( Figure 8a shows this operation for the example illustrated in Figure 7a), then to select one of the parts for putting ones in it (as shown in Figure 8b). According to counting the number of ones in this part and difference with number rows (shown in Figure 8b this value is one), another part is selected from another side the main diagonal of matrix with one element (place (1,3) as shown in Figure 8c).

**Figure 9:**
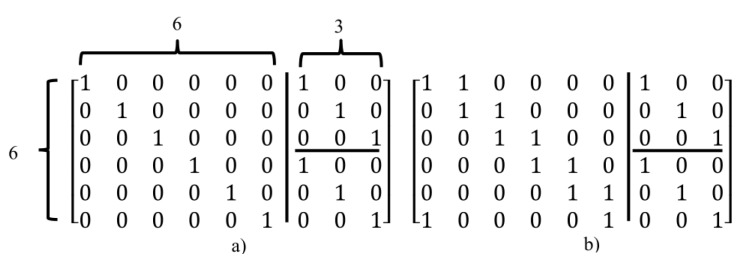
Creating a parity check matrix using method 1 (**a**) Step 1: put ones on the main diagonal, (**b**) Step2: put reminder ones as triangular state.

In general, for constructing an m×n (*m* show the number of rows and *n* show the number of columns) regular LDPCM with $w_{c}$ and $w_{r}$ as the non-zero elements in each column and row, first, the number of columns (n) is divided by the number of rows (m). The result is two matrices with (m×m) and (m×(n−m)) dimensions. Then, for the created square matrix (m×m) the aforementioned proposed method can be used. Another matrix can be converted to square matrices with a lower dimension $\left(\frac{m}{2}\times \frac{m}{2}\right)$. Then by using the above method again, ones are placed in needed places. For example, the parity check matrix for (n=9,wc=2,wr=3) code constructed by the first proposed method is given as shown in Figure 9.

**Figure 10:**
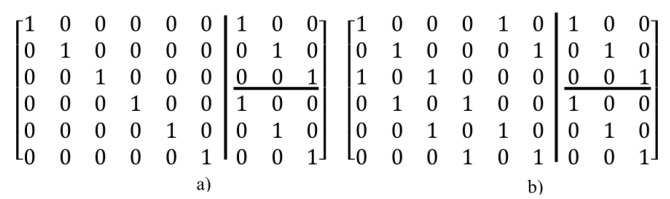
Another state for placing ones in regular Low-Density Parity-Check Matrices (LDPCM). (**a**,**b**) show step by step this method. (**a**) first step, (**b**) second step.

As shown in Figure 9, the main matrix is converted to a 6×6 matrix and two 3×3 matrices. First, ones are placed in the main diagonal for the three above matrices, then according to the number of ones needed for the columns of 6×6 matrix, elements are filled as triangular. The above method can certainly be used for the secondary diagonal elements or as a combination of both. Moreover, in this method, to determine the number of ones needed for each column, the number of them in each row is created. Furthermore, the above method can be used for each of the diagonal elements of the matrix as triangular. Figure 10 illustrates another state of Figure 9.

### 2.2. Second Method: Algebraic Construction

The second method is based on the conversion of the main matrix to smaller square matrices with $\frac{m}{f}\times \frac{m}{f}$ dimensions in which *m* is the number of rows and *f* is each number divisible to *m*. Then, for each of the created square matrices, the method proposed in the prior sub-section can be applied. For example, for creating a matrix with specification created in Figure 9, by using the second method, first, the main matrix must be converted to square matrices with lower dimensions. For example, dimensions of square matrices are $\frac{6}{2}\times \frac{6}{2}=\frac{3}{3}$ in which the number of square matrices is equivalent to the number of all elements divided by the dimension of square matrix elements $(\frac{6\times 9}{3\times 3}=6)$. Therefore, the created matrix is as Equation (12).

(12) $$\left[\begin{array}{ccc} A_{0} & A_{1} & A_{2}\\ A_{3} & A_{4} & A_{5} \end{array}\right]$$

**Figure 11:**
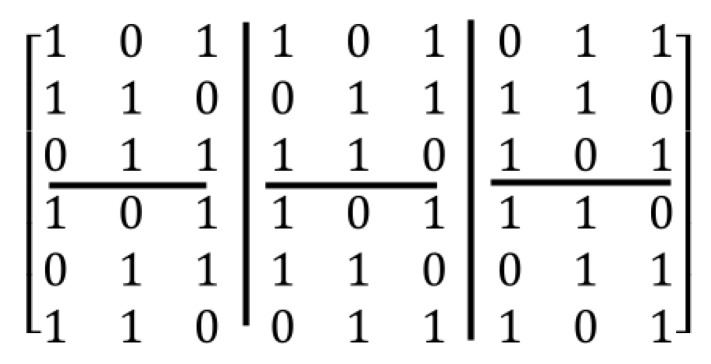
Representation of second method for matrix shown in Figure 9.

In which, $(A_{0}$ to $A_{5})$ are square matrices and each of them is created by the method mentioned in the previous section. For example, one of the states for Equation (12) is shown in Figure 11.

As shown in Figure 11, in this example, square matrices are created by the first method.

### 2.3. Post-Processing Method

For arriving to all possible states in the construction of regular parity check matrix $\left(n,w_{c},w_{r}\right)$, a post-processing method is used. In this method, after creating of primary parity check matrix, new states of the matrix can be created by using the two following operations:

- Column operations: in this method, for creating of a new state of the matrix, for the specified two columns, the value of one in a column is exchanged with the value of zero of another column and vice versa, i.e. this operation for the value of one in a column and value of zero in other column is done.
- Row operations: in this method, for creating a new state of the matrix, two rows are selected and then, the value of one in a row is exchanged with a value of zero in another row and vice versa.

**Figure 12:**
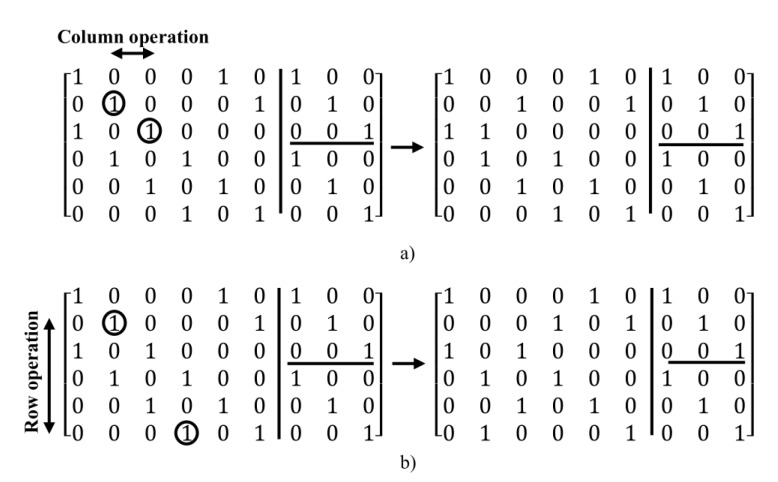
Illustration of applying (**a**) column operations and (**b**) row operations to matrix generated in Figure 9.

For example, in Figure 12, the two above operations are applied to the matrix created in the Figure 10.

As shown in Figure 12, by combining the row and column operations, linearity can arrive at all possible states for a certain matrix. In the next section, the post-processing method is used for removing the girth 4 in regular parity check matrices.

## 3. Proposed Algorithms for Removing the Girths 4 and 6

In this section, two methods for constructing the parity check matrix with removal of girths 4 and 6 are proposed.

### 3.1. Algorithm 1 for Removing Girth 4

**Figure 13:**
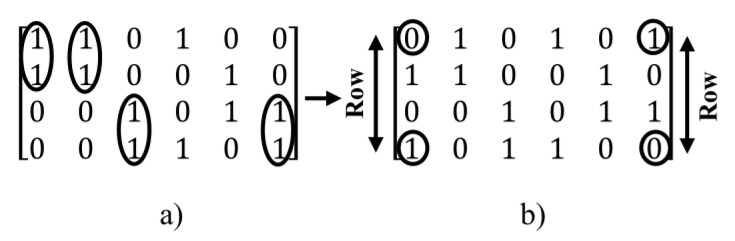
An example of removal of girths 4. (**a**) Finding girths 4, and (**b**) deleting girths 4 by the post-processing method.

In this method, the proposed post-processing method introduced in Section 2 is used for removing girths 4. Initially, the parity check matrix is created by one of the methods introduced in Section 2. Then, Tanner graph or lemma used in [22] can be applied for finding girths 4 in the matrix. This lemma states a girth 4 free matrix H as: if and only if all the entries of the matrix $H_{T}H$ are 1’s (ones) except the diagonal line. After the detection of girths, to apply Row and Column operations introduced in the post-processing method in specified places, the girths 4 are removed and a regular LDPC matrix is created without girths 4. This work is done until all cycles with the intended length are removed. For example, this method is shown in Figure 13.

### 3.2. Algorithm 2 for Removing Girth 4 and 6

#### 3.2.1. Algorithm 2-A: Without Girths 4

**Figure 14:**
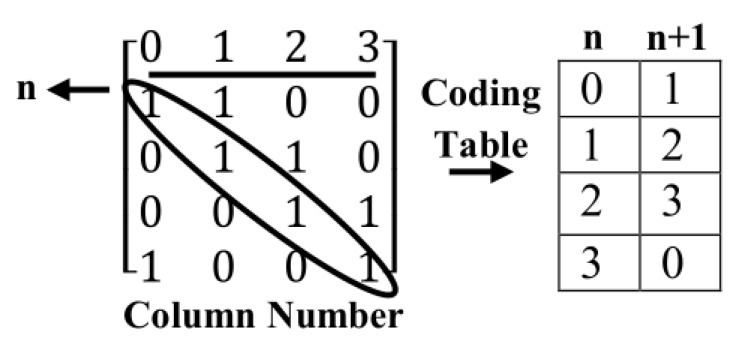
Illustration of coding matrix in second proposed method.

In this method, the Sarrus-based method is used for creating a parity check matrix without girth 4 with the following coding method as shown in Figure 14.

**Figure 15:**
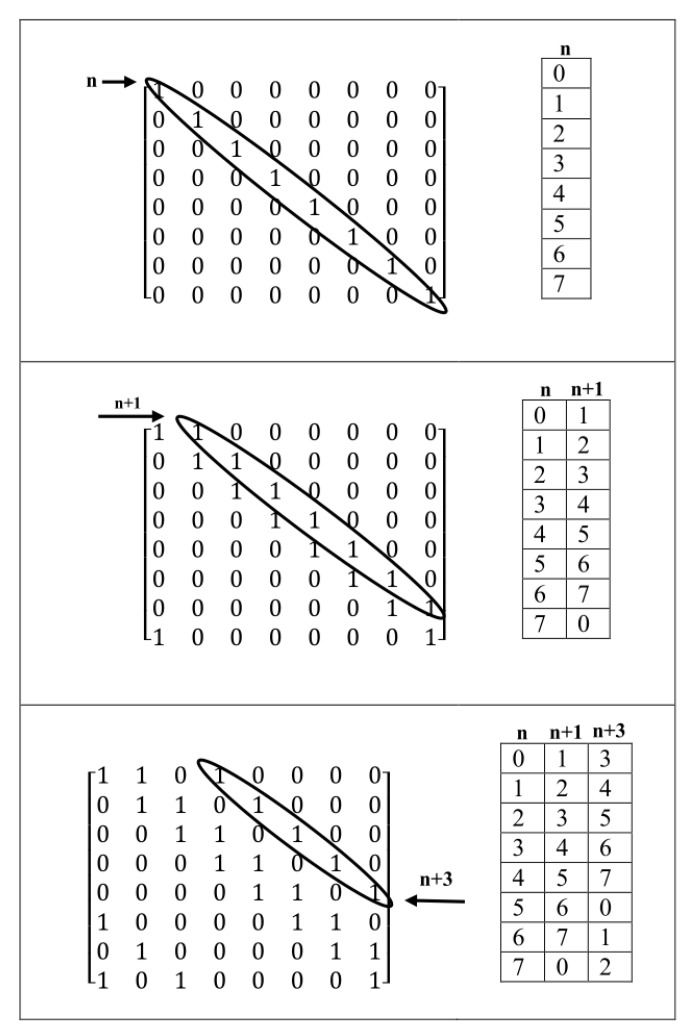
Creating of parity check matrix 8 × 8 using method 2 without girths 4.

Place of ones in the matrix is coded as column number or place of each one can be stated as shifted to the right into the first column of the left side and its number is placed in each row related to each one in a coding table. As shown in this Figure 14, the first column of the coding table shows the number of columns (rows) ones placed in the main diagonal of the matrix. This work is done for each one added as diagonal according to the Sarrus-based method. Ones in each additional diagonal are coded in an additional column in the coding table. Besides, the ones placed in the main diagonal can be stated as variable *n* and ones started from column 1 that as diagonal placed in matrix, can be stated as the distance into main diagonal (n+1). Generally, this work can be done for each of the ones placed as diagonal into the main diagonal of the matrix. For creating a parity check matrix without girths 4, each two one’s in each row must not have overlap with another row. In other words, each two one’s in each row, only once meet together in each two-column of coding table. So, as mentioned above, for creating a matrix n×n without girths 4, the value of variable related to column is checked. If a column has no overlap with variables related to another column, then the ones are placed diagonally. For example, in Figure 15, the first, ones are placed in the main diagonal.

So, the place of these ones is put in the first column (column 0) of the coding table and is represented by variable *n*. Next, the ones can be placed diagonally from column 1 of the matrix. These ones are symbolized by variable n+1. These two columns have not any overlap together. As in the coding table, the place of ones in these two columns is different and the distance of each place with other is ((n+1)−(n)=1). Then ones must be placed from column 3 as diagonal (n+3) and they cannot be placed from column 2. Because these columns have overlap with the first two columns. i.e., the difference between columns 1 and 0 is (n+1)−n=1. The difference between columns 2 and 1 is (n+2)−(n−1)=1. Thus, column 2 has an overlap (same difference) with two other columns. However, column 3 has two differences with column 1 and three differences with column 0, also, as column 1 (3) with a more velocity into Column 0 (1) increases. These columns are complementary faster. So, the difference of complementary of column 1 with 0 is (8 − 1) − 0 = 7 and differences of complementary of column 3 by column 1 and 0 are six and five. So these differences are various and have not any overlap. So, as shown in Figure 15, we can add ones as diagonal to column 3. By adding new ones as diagonal to another column is created an overlap. For example, Column 4 to Column 3 creates an overlap with columns 0 and 1. Columns 5 to column 3 creates an overlap with columns 3 and 1. Columns 6 to column 3 also create an overlap with columns 0 and 3. In final, complementary column 7 with 0 have overlap with columns 1 and 0 and all of these cases generate cycles with length 4. So, the maximum number of ones can be placed for creating regular parity check matrix (8×8) without girths 4 is three.

**Figure 16:**
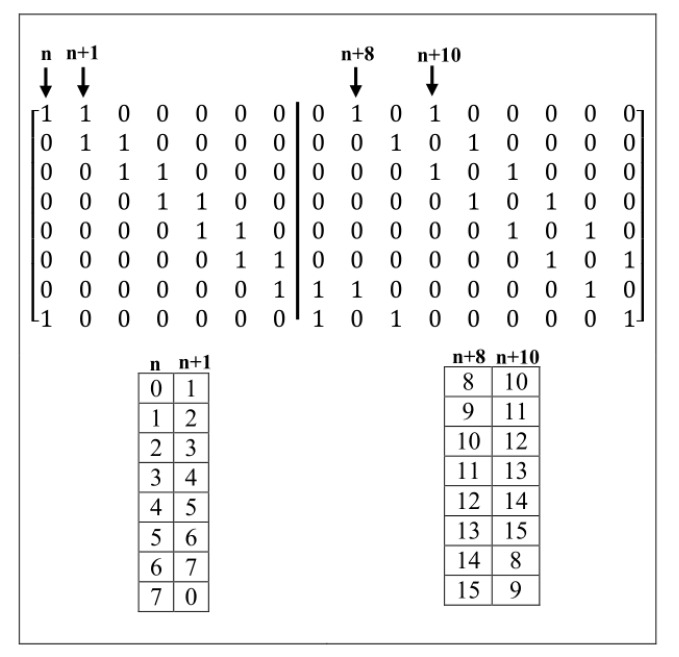
Using of the second method for creating matrix 8×16 without girths 4.

A matrix m×n, for example, 8×16, with $w_{c}=2$, can be created as shown in Figure 16.

First, according to Sarrus-Based method, the matrix is divided into two square matrices 8×8, then, ones are placed according to the coding table for each matrix. By using these two coding tables, girths 4 within each matrix and common girths 4 in two matrices are checked. As shown in Figure 16, as $w_{c}=2$, so the number of ones in each row of the matrix 8×8 must be two. In the second matrix 8×8, ones have been placed as diagonal from columns n+8 and n+10. i.e., in the second matrix, the numbers between 0 to 7 (column number) are shifted from 8 to 15. Differences between columns within the first matrix are one and seven. That difference seven is created from the difference of the second column complementary and the first column in the first matrix. In the second matrix, differences between columns are two (n+10)−(n+8)=2 and |(8−10)+8|=6 where the second number is the difference between the second columns complementary in the first column. For creating complementary in the second matrix, the number of columns is subtracted from eight. i.e. 8-(number of columns). Previously, the complementary column is summed with another column. Differences of columns between two matrices are 8, 7, 10, 9, 15, and 1 where two differences 15 and 1 are related to the difference between complementary of the second column of the first matrix and the first column of the second matrix (as n+8 is equaled with *n* by shifting to right to size eight, so column n+1 of the first matrix is complementary faster than another ((8 − 1) + 8 = 15)) and the difference between complementary of the second column of the second matrix and the second column of the first matrix (as the second column of the second matrix, n+10, is equaled to n+2 with shifted to the right to size eight, so, column n+10 is complementary faster into column n+1 i.e., |((8−10)+1)|=1), respectively.

#### 3.2.2. Algorithm 2-B: Without Girths 6 and 4

For creating a parity check matrix without girths 4 and 6, in addition, the before relation, the following relation must be established in the coding table. In the coding table, if the difference two columns are *d*, then two columns are found that differences in each of them into two other columns be *m* and *n*. Then there are girths 6 in matrix if the Equation (13) is established:

(13) d=m+n,ord=m−n.

**Figure 17:**
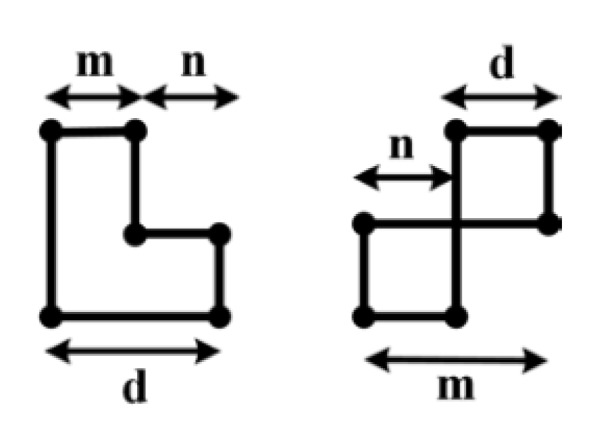
Illustration of general states of girth 6 in parity check matrix.

Equation (13) is according to general states of girth 6 in a parity check matrix as shown in Figure 17.

For example, consider matrix 8×8 generated in Figure 15. As stated above, this matrix with having three ones in each row have not any girths four. However, this matrix has girth six. Because column n+3 in the coding table, create an overlap according to Equation (13) with columns 0 and 1.

For example, columns 0 and 1 have a difference of one. Difference of Column 3 with 1 is two and if this value is subtracted from the difference of columns 0 and 1, it is equal to the difference of columns 0 and 1. i.e., (3 − 1) − (1 − 0)=1. Therefore, in this matrix, column 3 creates girths six. With deleting ones from this column, the matrix 8×8 has not any girths six. For creating a regular parity check matrix 8×8 without girths 4 and 6, this matrix can have a maximum of two ones in each column by using the Sarrus-based method. Generally, the method proposed for removing girths 4, create girths 6 for the number of the ones more than two. As distance of third columns from two other columns is the same and so, Equation (13) always is true for the number of three and more ones in the matrix.

**Figure 18:**
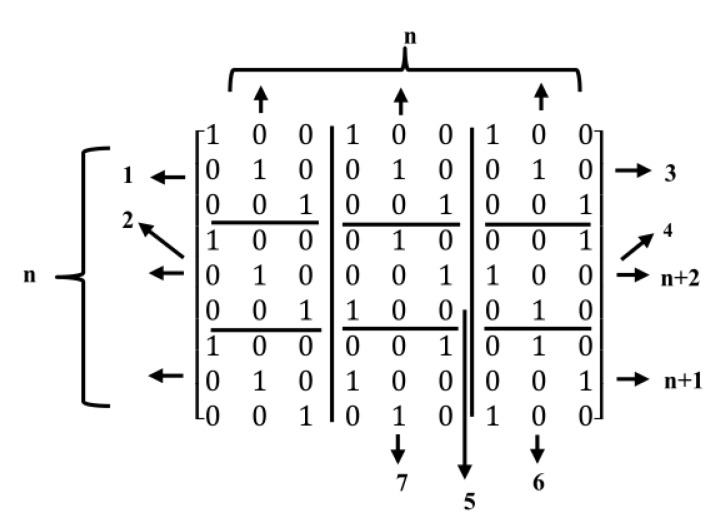
Illustration of the proposed method for creating matrix without girths 4 and 6.

For creating parity check matrix without girths 4 and 6, the algebraic construction method introduced in Section 2 is used as the following:

Consider a matrix m×m so that *m* is divisible to $w_{c}$. Then, the main matrix can be divided into $w_{c}$ square sub-matrix. Next, for each sub-matrix coding table as stated in the previous sub-section and Equation (13) is used. For example, consider a matrix 9×9 using this method without girth 4 and 6 as shown in Figure 18.

As shown in this figure, the first, sub-matrices of the left corner and top of the main matrix are filled with ones from column zero (n). Other matrices are filled with other states and according to the coding table so that for each four sub-matrices that two to two placed in the same row and same column, the method explained for creating matrix without girths 4 and Equation (13) must be satisfied.

For instance, consider sub-matrices 1, 2, 3, and 4. Differences of sub-matrices 1 and 3 (distance is zero) must not be the same with differences of sub-matrices 2 and 4 (distance is two). Furthermore, this feature also must be existed between sub-matrices 4, 5 with submatrices 6, 7 i.e., the difference between sub-matrices 4 to 5 is one but the difference between sub-matrices 6 to 7 is minus one (–1). Thus, the above features are satisfied.

## 4. Simulation Results and Analysis

**Figure 19:**
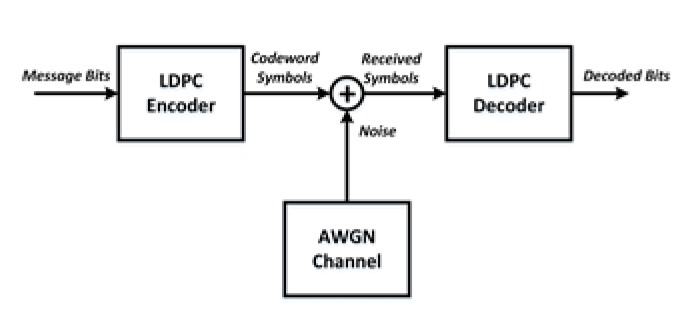
Illustration of LDPC encoding and decoding in additive white Gaussian noise (AWGN).

In this section, the proposed methods for generating regular parity check matrix without girth 4 and 6 are considered for the simulation. The proposed matrices have been simulated by using the sum-product algorithm(SPA) decoding [43,44] under the additive white Gaussian noise (AWGN) channel as shown in Figure 19.

The a priori information [43,44] for binary symmetric channel of the decoder (log likelihood ration (LLR)) is defined by:

(14) $$\gamma =\log\frac{Pr(x=0|y)}{Pr(x=1|y)}$$

The received binary sequence y in terms of binary phase shift keying (BPSK) modulation is given by:

(15) $$y=\left(1-2x\right)+n_{0}$$

Here, $n_{0}$ is the binary-input additive white Gaussian noise (BI-AWGN). The initial LLR, say γ [43,44], for the noise variance $\sigma^{2}$ is obtained as follow:

(16) $$\gamma =\frac{2}{\sigma^{2}}y.$$

From the a priori information obtained through the channel to the decoder, posterior information is calculated through the iterative procedure and then a hard decision is made for each posterior information until a valid code-word is found or the maximum iteration(Imax) (Imax=20 for example 1 and Imax=10 for examples 2 and 3) is reached.

**Figure 20:**
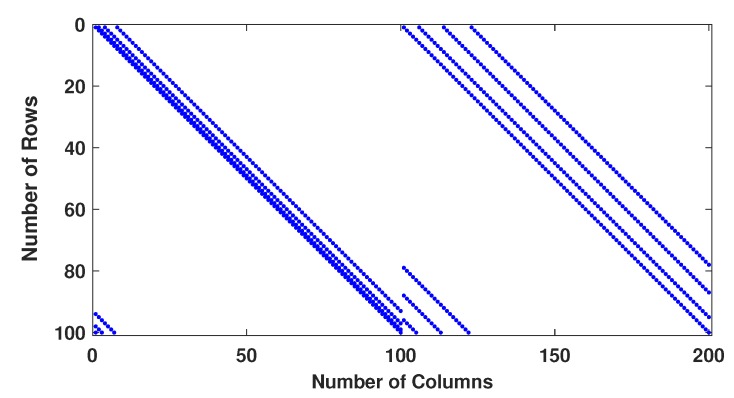
Distribution of 1’s in PCM (100, 200, 4, 8) generated by method 1.

**Figure 21:**
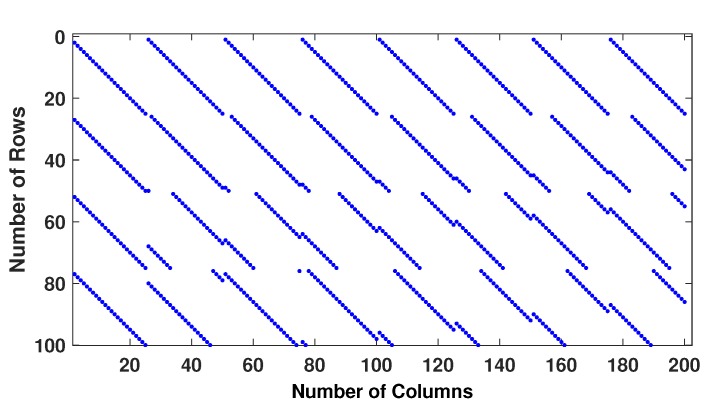
Distribution of 1’s in PCM (100, 200, 4, 8) generated by method 2.

**Figure 22:**
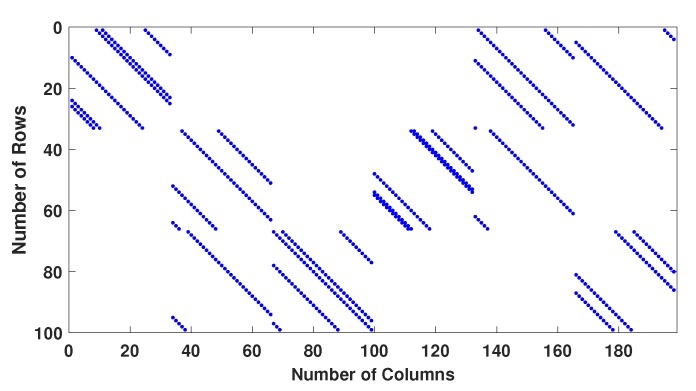
Distribution of 1’s in PCM (100 × 200) generated by QC PCM-TypeIII.

**Figure 23:**
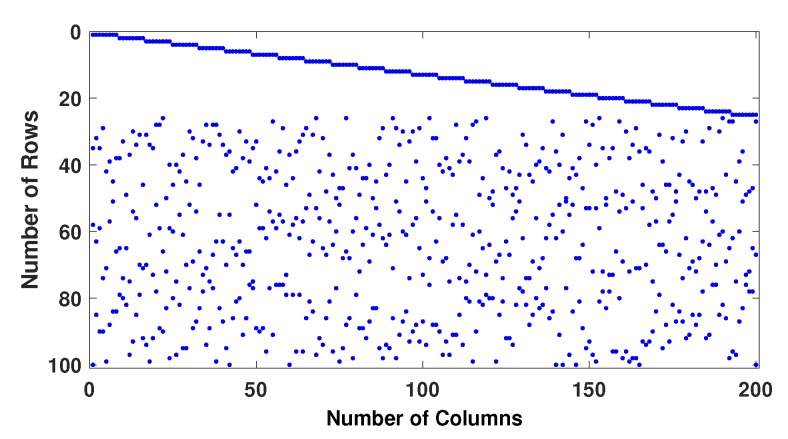
Distribution of 1’s in PCM (100, 200, 4, 8) generated by Gallagar’s method.

**Figure 24:**
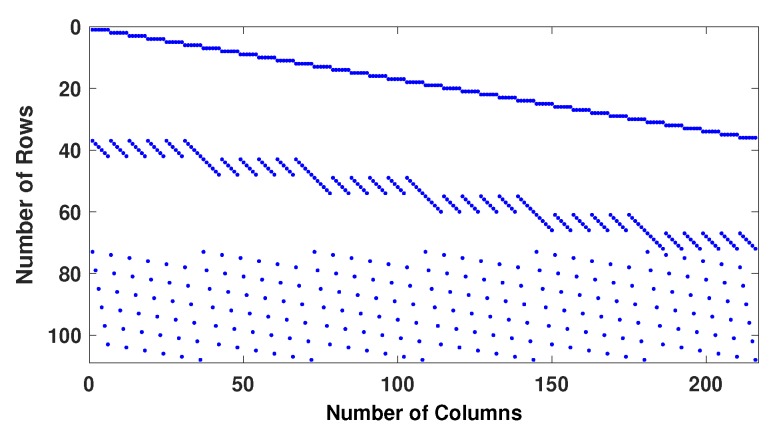
Distribution of 1’s in PCM (208,216,3,6) generated by J.Fan et al.

Example−1: Two regular parity check matrices with parameters $\left(m=100,n=200,w_{c}=4,w_{r}=8\right)$ have been created by proposed methods 1 and 2 and are ensured to be free of four cycles by use Algorithm 2-A. The parity check matrices created by method 2 is used to make sure the PCM is free of six cycles by use Algorithm 2-B. A randomly generated message bits (k=100) has been encoded with generator matrices produced by these specially designed parity check matrices (100, 200, 4, 8). A code-word (200, 8, 4) has been modulated as BPSK(+1 corresponds to 0 and -1 corresponds to 1) and transmitted over a binary input AWGN (BIWGN) channel. A performance comparison has been made with the standard quasi-cyclic (QC) parity check matrices (PCM) produced by methods known as type III and circularly shifted identity matrices [17,45,46]. In all these codes, the number of 1’s in the rows and columns are 8 and 4, respectively, while the length of the codeword is 200 for all except for method in [46]. The graphs in Figure 20 and Figure 21 show the distribution of 1’s in the parity check matrices( PCM) constructed by proposed methods 1 and 2 in this paper and QC matrices in literature are shown in Figure 22, Figure 23 and Figure 24. From the figures, we see that the distribution of 1’s in the proposed methods is systematic, hence results in improved performance as well as contribute towards efficient memory implementation.

**Figure 25:**
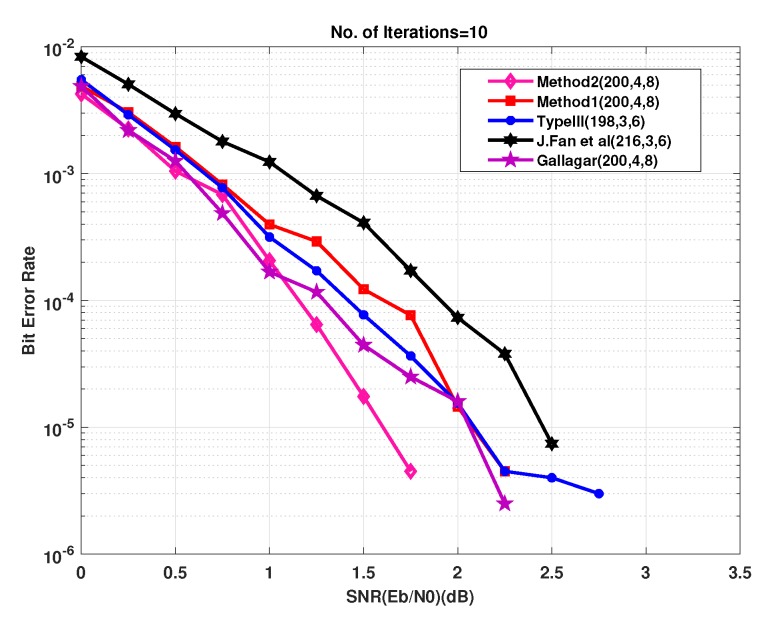
Bit error rate performance of parity check matrices.

Analyzing the graphs in Figure 25, from 0db to 1db, the performances of all the parity check matrices are almost similar and follow the same trend lines. The graph shows it clearly that after 1db, method 2 outperforms than all the matrices and even better than method 1. At 1.5db, method 1 has a gain of approximately 1db over the matrices created by J.Fan et al. method and has more than 0.5db gains over the other matrices. Method 2 performs better than J.Fan et al. method for all the SNR values.

The graph in Figure 25 illustrates the performance curves of the short length matrices created by the proposed methods and existing matrices in literature. From the graph, we see that for short length matrices, the performance of proposed matrices matches or performs better than the existing methods of the construction of the parity check matrices. The simulation results show the improved performance of these newly developed methods for the design of parity check matrices.

**Figure 26:**
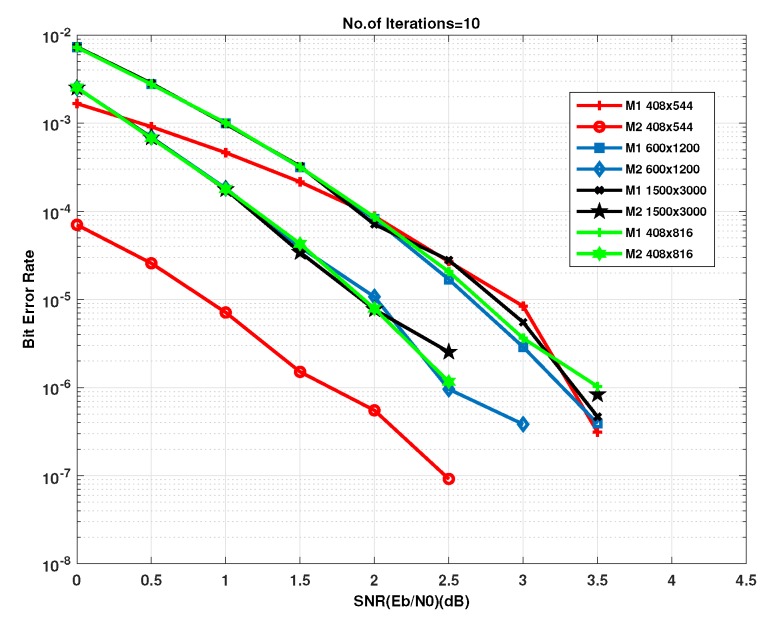
Bit error rate performance of parity check matrices.

Example-2: In this example, all zeros code-word has been selected to transmit and decode it using the matrices created by method 1 and 2 to compare the performances for different code length and code rate. Figure 26 shows the bit error performance curves for code $\left(408,544,w_{c}=3,w_{r}=4\right)$ with rate =3/4 and codes $\left(408,816,w_{c}=4,w_{r}=8\right),\left(600,1200,w_{c}=4,w_{r}=8\right)$ and $\left(1500,3000,w_{c}=4,w_{r}=8\right)$ of half rate has been chosen. The graphs in red colors show the codes created by method 1 and the graphs in black color shows the codes created by method 2. The graph in Figure 26 shows that the LDPC decoder outperform for the parity check matrices created by method 2 $\left(408,544,w_{c}=3,w_{r}=4\right)$. For all the SNR values, method 2 $\left(408,544,w_{c}=3,w_{r}=4\right)$ outperforms than all the other matrices. The coding gain of method2 $\left(408,544,w_{c}=3,w_{r}=4\right)$ at 1.5db is almost 1db from the codes of length 1200,3000,816 and approximately 2db from codes of length 544,816,1200,300. We see in this graphs that method 2 is more efficient and shows high performance than method 1.

**Figure 27:**
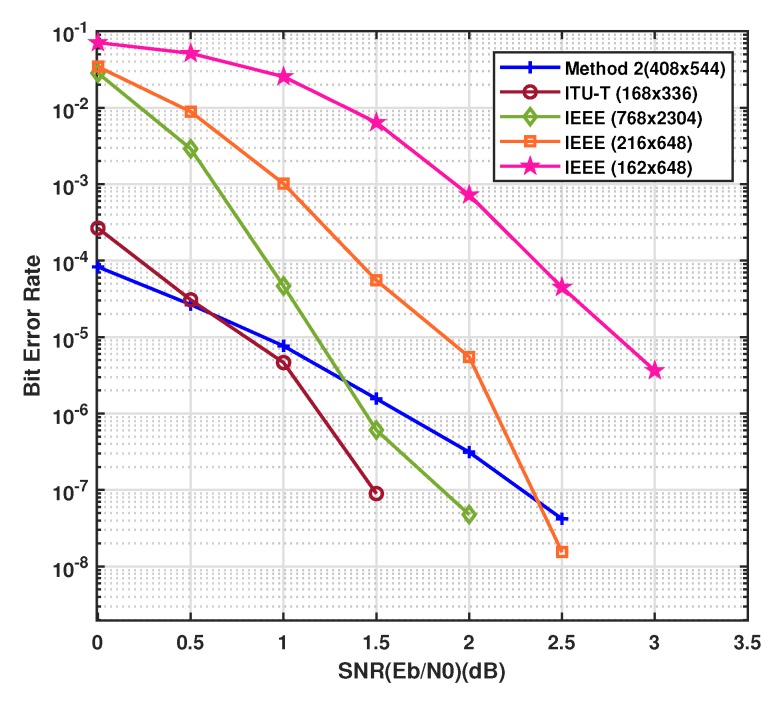
Bit error rate performance of parity check matrices.

Example-3: In this example, the parity check matrix constructed by the proposed method 2 has been simulated to compare the BER performance with standard parity check matrices like IEEE and ITU-T [38]. An IEEE802.16-2009 LDPC code of parity check matrix 768 × 2304 with code rate 2/3 is simulated along with IEEE802.11-2012 codes of parity check matrices 216 × 648 and 162 × 648 with code rates 2/3 and 3/4 respectively as shown in Figure 27. The ITU-T G.9960 standard LDPC code of length n=336 with code rate=1/2, have been simulated to compare the results with the proposed method 2 as mentioned above. From the curves in the graph shown in Figure 27, we see that the proposed method 2 is performing close to the ITU-T and IEEE802.16-2009 standard while IEEE802.11-2012 LDPC code of size 162 × 648 is less in performance than all the methods simulated in the Figure 27. A noticeable performance gain of the proposed parity check matrix can be seen in the Figure 27 between 0db and 0.5db over IEEE802 and ITU-T.

The graph also shows the high performance of the proposed methods for creating a moderate length of LDPC codes. These codes are particularly suitable where information length is small or medium. On the other way, when the information length varies from small to large length as in the case of IoT and 5G and beyond mobile communication networks, the parity check matrices created by the proposed methods are of special interest and advantage over all others.

### EXIT Chart Analysis

**Figure 28:**
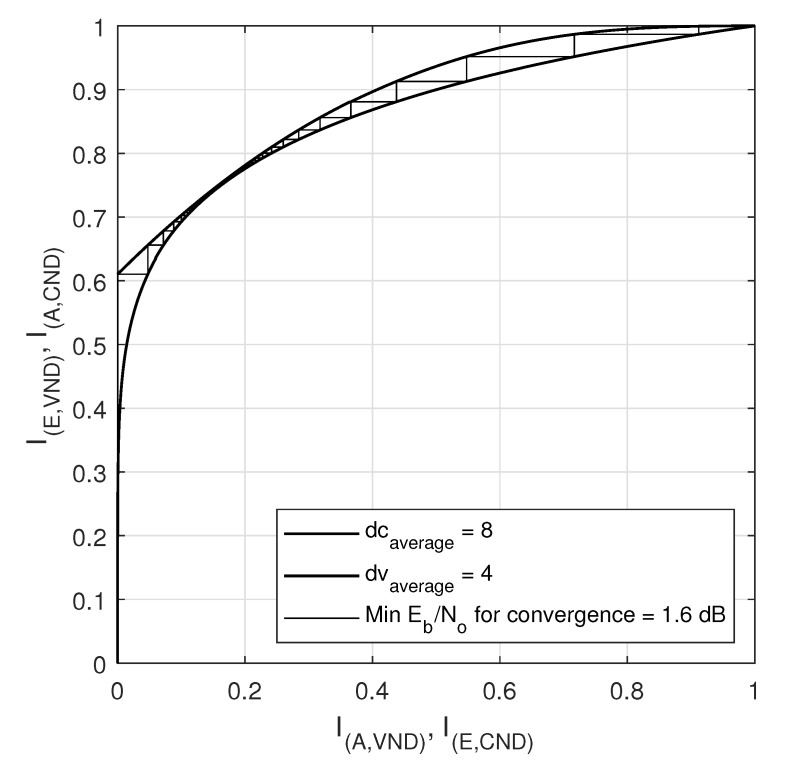
EXIT curve for LDPC code (816,4,8) with rate = 0.5.

**Figure 29:**
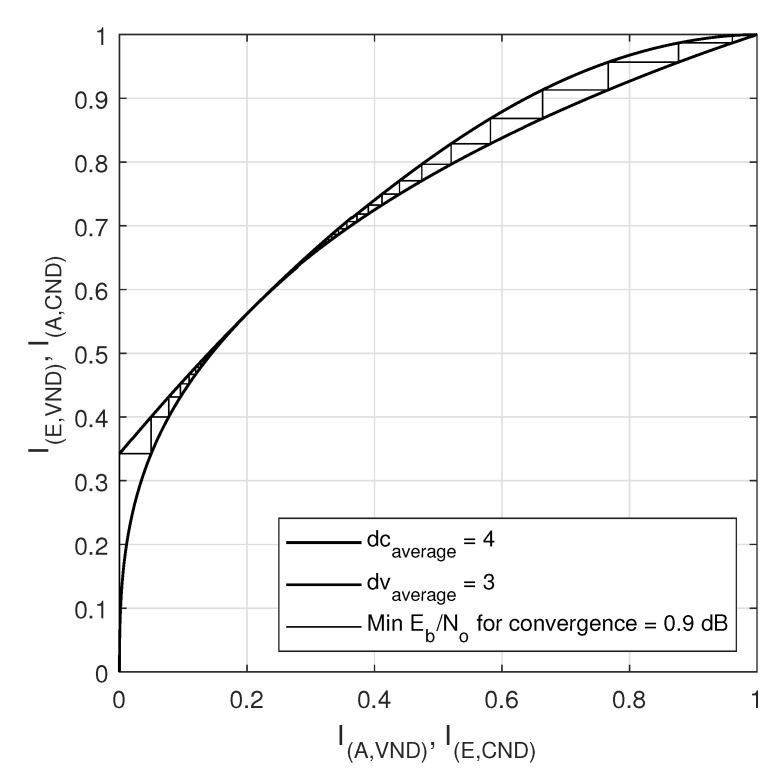
EXIT curve for LDPC code (544,3,4) with rate = 0.25.

Extrinsic-information-transfer (EXIT) chart is a graphical tool that aids in the estimation of decoding thresholds of LDPC codes. EXIT charts utilize Gaussian approximations to provide information on the dynamics of convergence properties of an iteratively decoded code [38]. EXIT charts are based on the principle that the variable node and the check-node work iteratively to reach the convergence [47]. A plot for the transfer curve based on the input information versus the output information can be attained for the VN and for the CN, where the transfer curve for the variable node processor depends on the SNR in the channel. The iterative decoding behavior is represented by a staircase function in the space between the two curves of the VN and CN. The EXIT charts have been plotted for two regular LDPC codes (408, 544,wc = 3,wr = 4) and (408, 816,wc = 4,wr = 8), constructed by the proposed method 2, as shown in Figure 28 and Figure 29 respectively.

**Figure 30:**
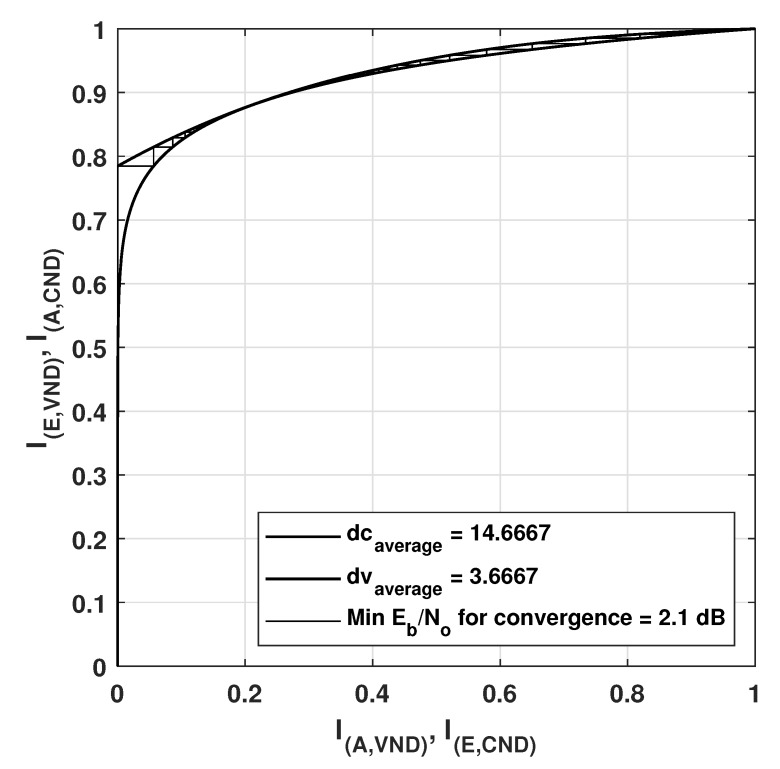
EXIT curve for IEEE802.11-2012 LDPC code with paritych check matrix of size 162 × 648 with rate = 0.75.

$I_{\left(A,VND\right)}$ is the a priori mutual information of variable nodes input (plotted on x-axis) and $I_{\left(E,VND\right)}$ is the extrinsic mutual information is the extrinsic or output mutual information of variable nodes (plotted on y-axis). While $I_{\left(A,CND\right)}$ is the a priori mutual information of check nodes input part (plotted on y-axis) and $I_{\left(I,ECND\right)}$ is the extrinsic mutual information or output mutual information of check nodes (plotted on x-axis). In the Figure 28 and Figure 29, the minimum convergence is listed as 0.9db and 1.6db for the LDPC codes with a code rate of 0.25 and 0.5 respectively. Figure 30 shows the EXIT chart for the IEEE 802.11-2012 LDPC code with a parity check matrix of size 162 × 648 and code rate 0.75.

## 5. Conclusions

The proposed methods of generating low-density parity-check matrices are presented to enhance the decoding performance of the LDPC iterative algorithms to ensure the data integrity of the social IoT in the presence of noise. The simulation results demonstrate that the medium and short length codes constructed by the proposed methods perform well over AWGN channels using the iterative LDPC sum–product decoding algorithm. We see that the sparse matrices designed by the proposed methods show better decoding performance than the existing methods for parity check matrices of short and moderate length. It can also be seen that even for a small number of iterations, the BER performance of the LDPC decoder is much better for the proposed parity check matrices. The cyclic structure and better performance for short and medium lengths codes of the proposed methods are making them suitable for hardware implementation and practical application like IoT and mobile networks where data reliability and integrity are the main challenges.
